# Angioleiomyoma of the Foot Presenting as a Ganglion Cyst: A Case of Mistaken Identity

**DOI:** 10.7759/cureus.90980

**Published:** 2025-08-25

**Authors:** Jochen Gerstner Saucedo, Natalia Coriat, Yudel Tamayo, Jorge Moreno, Juan B Gerstner, Fabiano N Cardoso

**Affiliations:** 1 Diagnostic Radiology, University of Colorado Anschutz Medical Campus, Aurora, USA; 2 General Surgery, Clinica Imbanaco, Cali, COL; 3 Diagnostic Radiology, University of Miami Miller School of Medicine, Jackson Memorial Hospital, Miami, USA; 4 Pathology, Clinica Imbanaco, Cali, COL; 5 Orthopedics and Traumatology, Clinica Imbanaco, Cali, COL

**Keywords:** angioleiomyoma, benign smooth muscle tumor, foot, ganglion cyst, soft tissue mass

## Abstract

Angioleiomyoma is an uncommon benign neoplasm originating from the smooth muscle of vascular walls, typically presenting as a painful subcutaneous mass in the lower extremities of middle-aged adults. However, atypical features such as painless growth and unusual anatomical locations can obscure the diagnosis, particularly when limited imaging modalities are used. We present the case of a 62-year-old woman with a 10-month history of right ankle discomfort and a recently enlarging, painless soft-tissue mass over the anterior aspect of the ankle. Radiographic evaluation revealed a subtle soft-tissue prominence adjacent to the first tarsometatarsal joint, without calcifications or osseous involvement. Based on the clinical presentation and radiographs, a ganglion cyst was presumed, and the patient proceeded to surgical excision at her request to relieve symptoms and avoid delays. Intraoperatively, a well-circumscribed, lobulated mass was found superficial to the anterior tibial tendon sheath. Histopathology confirmed a venous-type angioleiomyoma with classic smooth muscle and vascular features, and immunohistochemistry demonstrated strong smooth muscle actin (SMA) positivity. This case illustrates a diagnostic pitfall where reliance on clinical impression and plain radiographs alone may lead to misdiagnosis. It emphasizes the value of contrast-enhanced MRI or ultrasound for preoperative assessment of indeterminate soft-tissue lesions, even when benign pathology is suspected. To our knowledge, this is the first reported case of angioleiomyoma arising adjacent to the anterior tibial tendon insertion at the first tarsometatarsal joint, expanding the known anatomical spectrum of this rare tumor.

## Introduction

Angioleiomyoma is a rare benign tumor arising from the smooth muscle of vascular walls, most commonly found in the extremities of middle-aged adults with a slight female predominance noted in several series [1,2]. These tumors typically present as small, slow-growing subcutaneous nodules that are often painful; however, in atypical cases, pain may be absent. While most angioleiomyomas predominantly occur in the lower limbs, unusual anatomical locations and nonspecific clinical symptoms can obscure diagnosis, especially when imaging is limited to plain radiographs [3,4].

While plain radiographs are often the initial imaging modality for evaluating limb masses, their role in diagnosing soft-tissue tumors such as angioleiomyoma is limited. X-rays can aid in excluding osseous involvement and detecting mineralization or calcification that might suggest alternative diagnoses, including myositis ossificans, synovial sarcoma, or calcified lipomas. However, angioleiomyomas are typically radiographically occult or nonspecific, and calcifications, when present, are rare and not diagnostic [1,5,6]. In contrast, MRI and ultrasound offer superior soft-tissue characterization, vascularity assessment, and anatomic delineation, which are critical for narrowing the differential diagnosis and surgical planning [7-9]. Therefore, while radiographs remain useful in the initial assessment, they should not be solely relied upon for preoperative planning of indeterminate soft-tissue masses.

This case highlights the diagnostic uncertainty posed by a rapidly enlarging ankle mass initially presumed to be a ganglion cyst based solely on clinical and radiographic evaluation. The final diagnosis of angioleiomyoma was confirmed exclusively through histopathology, reinforcing the essential role of tissue analysis in evaluating soft-tissue lesions with indeterminate imaging features [10]. To our knowledge, this is the first reported case of angioleiomyoma most likely arising from a small subcutaneous or peritendinous vessel adjacent to the anterior tibial tendon insertion at the first tarsometatarsal joint. This uncommon anatomical origin, not previously described in large case series or systematic reviews [2], expands the recognized spectrum of foot and ankle angioleiomyomas.

## Case presentation

A 62-year-old female with no relevant medical history presented with right ankle pain that began 10 months prior, accompanied by the appearance of a soft-tissue mass in the same region. The patient reported that the mass had progressively increased in size over the previous days and is now causing discomfort when wearing shoes that cause friction with the location of the mass. On physical examination, a single, lobulated, and mobile mass was palpated over the anterior aspect of the right ankle, without tenderness. The mass appeared to originate at the region of insertion of the anterior tibial tendon.

**Figure 1:**
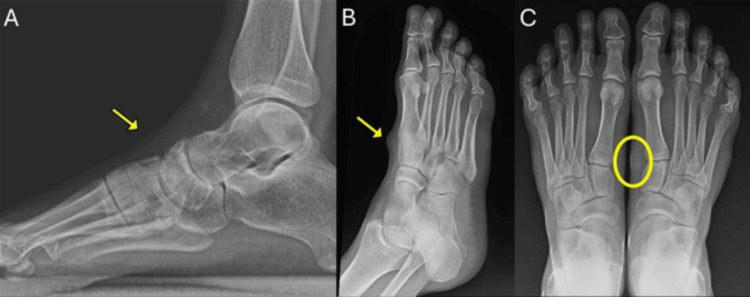
Weight-bearing lateral (A) and dorsoplantar oblique (B) radiographs of the right foot, and dorsoplantar view of both feet (C). The yellow arrows indicate the area of small soft-tissue mass with increased soft-tissue density adjacent to the right first tarsometatarsal joint. Image C highlights asymmetry between the right and left midfoot regions, with soft-tissue prominence on the right (circled). No evidence of bone involvement, periosteal reaction, or adjacent osseous destruction. No intra-articular abnormalities or calcifications were identified. The lateral radiograph was cropped to enhance visualization of the soft-tissue mass.

Weight-bearing lateral and dorsoplantar oblique radiographs of the right foot were obtained. The radiographs demonstrated a subtle soft-tissue mass with mildly increased density located adjacent to the first tarsometatarsal joint (Figures 1A-1C). There were no calcifications or evidence of bone erosion. Based on the physical examination and imaging findings, the initial suspicion was a ganglion cyst arising from the anterior tibial tendon sheath.

**Figure 2:**
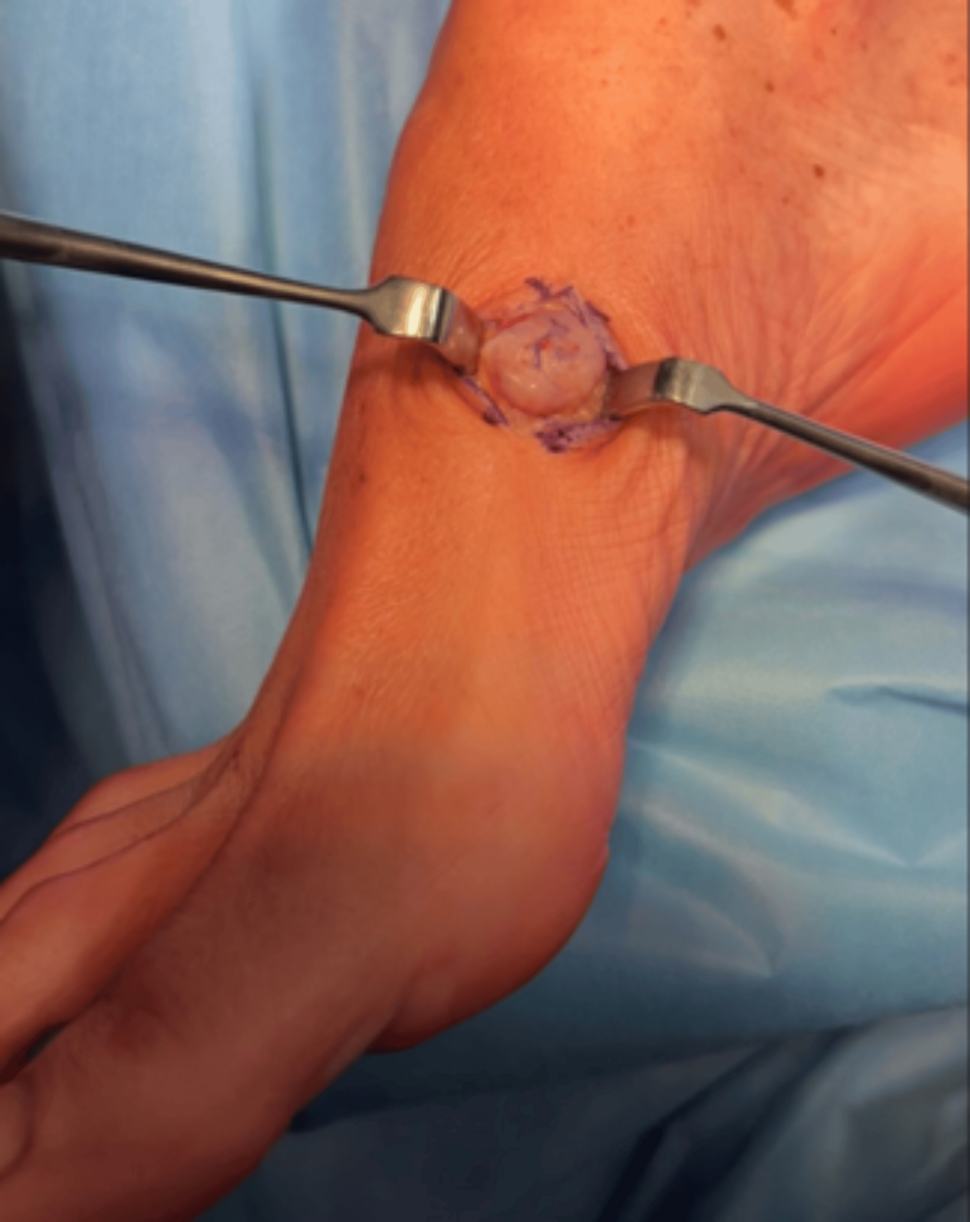
A longitudinal incision was made over the anterior aspect of the right ankle, centered over the palpable mass. Upon dissection, a well-defined, lobulated lesion was identified.

The patient expressed a strong desire to have the mass excised promptly due to increasing discomfort and interference with footwear. Given the lesion’s superficial location, benign clinical features, and radiographic appearance, the clinical team did not pursue additional imaging such as ultrasound or MRI, to avoid delaying surgical management. A tenosynovectomy and soft-tissue lesion resection were performed. Upon dissection, a well-defined, lobulated solid lesion was identified. The mass was located superficial to the anterior tibial tendon sheath and was carefully dissected from the surrounding soft tissues. The lesion appeared intact, with no evidence of capsular rupture, and was found adherent to or arising from the vascular structures within the anterior tibial tendon sheath (Figure 2).

**Figure 3:**
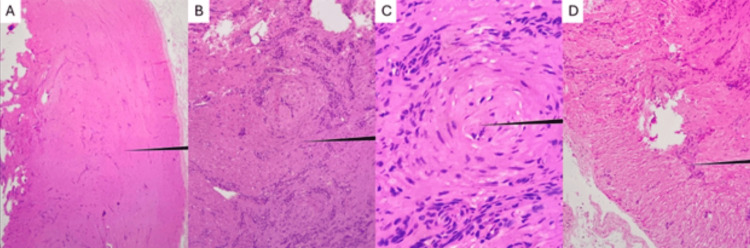
Histopathologic analysis of the excised lesion, stained with hematoxylin and eosin (H&E). (A) Low-power view showing a well-circumscribed, sharply demarcated lesion with features indicative of smooth muscle origin. (B) Medium-power view demonstrating prominent, thick-walled blood vessels with bland cytologic features. (C) High-power view highlighting a concentric (whorled) arrangement of smooth muscle fibers surrounding a vessel. (D) Additional medium-power field showing fascicles of elongated smooth muscle cells. Overall findings support the diagnosis of a benign angioleiomyoma.

Four weeks postoperatively, histopathological examination revealed a well-circumscribed, sharply demarcated lesion with features consistent with smooth muscle origin. Prominent thick-walled blood vessels with bland cytologic features were observed, surrounded by fascicles of smooth muscle cells arranged in a concentric (whorled) pattern around the vessels. These findings were consistent with a diagnosis of angioleiomyoma, specifically of the venous subtype, given the prominence and wall thickness of the vascular structures (Figures 3A-3D).

At her six-week follow-up visit, the patient reported complete resolution of symptoms, with no residual discomfort or limitation in footwear use. Physical examination showed a well-healed incision site without palpable mass or signs of recurrence. No postoperative complications were observed.

## Discussion

Angioleiomyomas are rare, benign tumors originating from the smooth muscle layer of vascular walls, accounting for approximately 4%-5% of all benign soft-tissue tumors. These lesions most frequently involve the lower extremities of middle-aged adults, predominantly among females. Clinically, they typically present as small, slow-growing subcutaneous nodules that are often painful; however, the pain is not a universal feature and appears to correlate with the lesion’s vascularity and histological subtype [3,4].

In this case, the patient presented with a progressively enlarging, painless mass over the anterior aspect of the midfoot, an uncommon location for angioleiomyomas. The absence of pain and the lesion’s superficial location over the tendon sheath led to a preoperative clinical suspicion of a ganglion cyst, a far more frequent benign lesion in this anatomical region. Imaging with plain radiographs revealed only subtle soft-tissue prominence, with no specific features suggestive of a vascular neoplasm. Such nonspecific radiographic findings are well-documented in the literature, emphasizing the diagnostic challenges posed by angioleiomyomas, particularly in cases where advanced imaging modalities such as magnetic resonance imaging (MRI) or ultrasonography are not performed [11,12].

In this context, the absence of cross-sectional imaging, while aligned with the patient’s desire for prompt resolution and the benign clinical appearance, represents a diagnostic pitfall. Although radiographs ruled out osseous involvement, they lacked the contrast resolution necessary to characterize the internal architecture or vascularity of the lesion. MRI and ultrasound are well-documented as superior modalities for evaluating soft-tissue masses, especially in differentiating solid from cystic components and assessing involvement of adjacent structures [7,8,13]. In this case, the reliance on clinical impression and plain radiographs alone led to an unexpected histopathologic diagnosis. This highlights the educational value of considering advanced imaging, even for seemingly benign lesions, when surgical excision is planned.

Although MRI was not performed in this case due to the initial clinical suspicion of a benign ganglion cyst and the patient's symptoms prompting expedited surgical intervention, it is worth noting the characteristic MRI features that may facilitate preoperative diagnosis when imaging is available. Edo et al. provided the most comprehensive description of these features in their large series correlating MRI findings with histopathology in angioleiomyomas [8]. They described the “dark reticular sign” on T2-weighted sequences, hypo- or iso-intense linear or branching structures within a hyperintense mass, most frequently observed in venous and cavernous subtypes. This sign, along with a peripheral hypointense rim and proximity to vascular structures, may help suggest the diagnosis, although these features are not pathognomonic [8].

Other studies have consistently reported that angioleiomyomas present as well-circumscribed, oval, subcutaneous lesions typically isointense to skeletal muscle on T1-weighted MRI sequences and heterogeneously hyperintense on T2-weighted or short tau inversion recovery (STIR) images. These lesions often demonstrate strong or heterogeneous enhancement following gadolinium administration and may contain internal linear or branching high-signal components that reflect vascular architecture [7,13,14].

The differential diagnosis for a subcutaneous soft-tissue mass with nonspecific findings on plain radiographs is broad and encompasses both benign and malignant entities. Among the most common benign lesions are lipomas, which typically present as soft, mobile, and painless masses. These lesions are characteristically radiolucent and may remain undetectable on radiographs unless they reach substantial size or undergo calcification [15].

Vascular anomalies, such as venous malformations and hemangiomas, may also present as soft, compressible masses and may occasionally display phleboliths; however, these features are rarely appreciated on standard radiographs [16]. Other benign lesions including epidermoid cysts and ganglion cysts typically appear well-circumscribed, slowly enlarging masses, with minimal or absent radiographic findings [17,18]. Nerve sheath tumors, such as schwannomas and neurofibromas, may present clinically similar, but are generally not visible on plain radiographs unless they are large [19]. Additionally, giant cell tumors of the tendon sheath, more commonly encountered in the hands and feet, can occasionally produce pressure erosions on bone, although they are generally not apparent on plain radiographs [20].

It is important to consider malignant lesions, such as soft-tissue sarcomas, malignant peripheral nerve sheath tumors, or metastatic disease, particularly in cases characterized by rapid growth, pain, neurological symptoms, or lesions larger than 5 cm in size. Certain radiographic findings, such as soft-tissue density, calcification, or bone involvement, may raise suspicion for malignancy; however, many sarcomas remain radiographically occult. Therefore, clinical judgment is essential, and further imaging or biopsy is necessary when malignancy cannot be confidently excluded [21,22].

In this case, the slow-growing, painless nature of the lesion and its superficial location over the anterior midfoot led to a preoperative clinical impression of a ganglion cyst, a much more common benign entity in this region. However, the final diagnosis of angioleiomyoma was established only after surgical excision and histopathological evaluation, revealing a diagnostic pitfall. This underscores the importance of maintaining a broad differential diagnosis, even when clinical and radiographic findings appear benign, and highlights the potential for misdiagnosis when cross-sectional imaging is omitted. Particularly in atypical cases, contrast-enhanced MRI or ultrasound may provide valuable preoperative clues, but definitive diagnosis ultimately rests on histopathology [2,23].

Histologically, angioleiomyomas are classified into solid (capillary), venous, and cavernous subtypes, with the solid type being most common in the extremities [8,24]. In this case, the lesion demonstrated prominent thick-walled blood vessels surrounded by fascicles of smooth muscle cells arranged in a concentric (whorled) pattern, findings most consistent with the venous subtype [8,24]. Immunohistochemical staining confirmed smooth muscle origin with strong positivity for smooth muscle actin (SMA) [3,24,25] . No cytologic atypia, necrosis, or mitotic activity was identified, consistent with the benign nature of angioleiomyoma, for which malignant transformation is exceedingly rare [2,26].

This case emphasizes the importance of including angioleiomyoma in the differential diagnosis of soft-tissue masses in the extremities, even when they occur in atypical locations or are asymptomatic. It also highlights the limitations of plain radiographs, which may not provide sufficient diagnostic information. In instances where imaging findings are inconclusive, histopathological evaluation remains essential for establishing an accurate diagnosis. Given the benign nature of angioleiomyomas and their excellent prognosis following complete surgical excision, timely recognition and surgical intervention are critical to achieving optimal outcomes.

## Conclusions

Angioleiomyoma remains an uncommon, benign soft-tissue neoplasm that is frequently misdiagnosed or overlooked in the preoperative setting due to its nonspecific clinical presentation and inconclusive radiographic features. This case highlights the diagnostic limitations of plain radiography, which often fails to provide definitive findings, and underscores the indispensable role of histopathological examination in establishing a final diagnosis. Although typically presenting as a painful, slow-growing subcutaneous mass, atypical painless cases such as this one should also prompt consideration of angioleiomyoma in the differential diagnosis of soft-tissue nodules, particularly in the extremities.

To avoid diagnostic pitfalls, clinicians are encouraged to consider ultrasound or contrast-enhanced MRI in the workup of soft-tissue masses that are ambiguous, atypical, or located in less common sites. Surgical excision remains both diagnostic and curative, with excellent prognosis and minimal risk of recurrence. Increased awareness of this entity and its variable presentation is essential to ensure timely diagnosis and appropriate management.
